# Prevention of bronchopulmonary aspiration in digestive surgery: the impact of training healthcare professionals

**DOI:** 10.1590/0102-672020260000050e1979

**Published:** 2026-10-05

**Authors:** Marcelo Gustavo PEREIRA, Guilherme Kenji Sacamoto GUIMARÃES, Franciele Ferreira BELIZARIO, Elaine Cristina ATAÍDE, Ivan Felizardo Contrera TORO, Ilka Fatima Santana Ferreira BOIN

**Affiliations:** 1 Universidade Estadual de Campinas, Faculty of Medical Sciences, University Hospital – Campinas (SP), Brazil.; 2 Universidade Estadual de Campinas, Faculty of Medical Sciences, Department of Surgery – Campinas (SP), Brazil.

**Keywords:** Health Education, Preoperative Care, Postoperative Care, Morbidity, Mortality, Educação em Saúde, Cuidados Pré-Operatórios, Cuidados Pós-Operatórios, Morbidade, Mortalidade

## Abstract

**Background:**

Bronchopulmonary aspiration occurs when food or fluids enter the lower airways, potentially causing hypoxia, aspiration pneumonia, and even death. Prevention requires early identification of risks and action by a multidisciplinary team. Continuous team education, using well established protocols, is essential to reinforce preventive measures and improve healthcare.

**Aims:**

Based on the well-established protocols, evaluate the impact of training on professional learning and the incidence of bronchopulmonary aspiration.

**Methods:**

A prospective, blinded, time-allocated, quasi-experimental study conducted in the gastrointestinal surgery ward and intensive care unit. The study was divided into three phases: patient assessment, multidisciplinary training based on the well-established protocols, and patient reassessment regarding risks, bronchopulmonary aspiration, and outcomes.

**Results:**

The study included 600 of the 710 patients evaluated, distributed in two phases: Phase 1 (n=267) and Phase 3 (n=333). Sixty-six professionals participated in Phase 2. The impact of training on bronchopulmonary aspiration was assessed through pre- and post-training assessments. The score increased significantly (pre-test: 75.06±13.36; post-test: 93.58±5.63; p<0.0001). In Phase 1, 32 patients presented with bronchopulmonary aspiration; in Phase 3, this number was 20, a significant difference (p=0.0097).

**Conclusions:**

Training proved effective in preventing bronchopulmonary aspiration and improving clinical outcomes, with a positive impact at all levels of the protocol employed. The results reinforce continuing education as a strategy for patient safety and risk management, suggesting its application in other healthcare settings.

## ARTICLE HIGHLIGHTS

Structured multidisciplinary training significantly improved healthcare professionals’ knowledge of bronchopulmonary aspiration prevention.

Systematic risk assessment identified bronchopulmonary aspiration risk in 86.8% of hospitalized patients.

The incidence of bronchopulmonary aspiration decreased by 50% after the training intervention, from 12.0% to 6.0% (p=0.0097).

Bronchopulmonary aspiration was associated with prolonged hospitalization and higher mortality, highlighting the importance of preventive strategies and continuous professional education.

## Summary

**CENTRAL MESSAGE:**

Bronchopulmonary aspiration is a significant cause of morbidity and mortality, particularly affecting three major demographic groups: children, older adults, and patients admitted to intensive care units. Individuals with dysphagia, patients in the postoperative period following digestive surgery, and those with neurological impairment are also particularly vulnerable to this event. The main risk factors include altered level of consciousness, reduced gastrointestinal motility, poor oral hygiene, invasive mechanical ventilation, enteral feeding tubes, prolonged vomiting, gastric obstruction, and advanced age. Other associated conditions include seizures, stroke, intoxication, pharyngeal muscle injury, ineffective cough, anatomical abnormalities, use of sedative agents, head and neck surgery, and changes in body position. Education of multidisciplinary healthcare teams and continuous professional training can facilitate the assessment and improvement of adherence to preventive measures and contribute to the effective prevention of bronchopulmonary aspiration.

**PERSPECTIVES:**

This study clearly demonstrated, with statistical significance, that structured multidisciplinary training is effective in preventing bronchopulmonary aspiration and improving clinical outcomes in a university hospital. The study primarily focused on patients with digestive system diseases. These findings reinforce the importance of well-designed educational interventions as a strategic tool for improving healthcare quality. Furthermore, they provide a strong foundation for implementing institutional policies focused on risk management and the prevention of adverse events.

## VISUAL ABSTRACT

**Figure:**
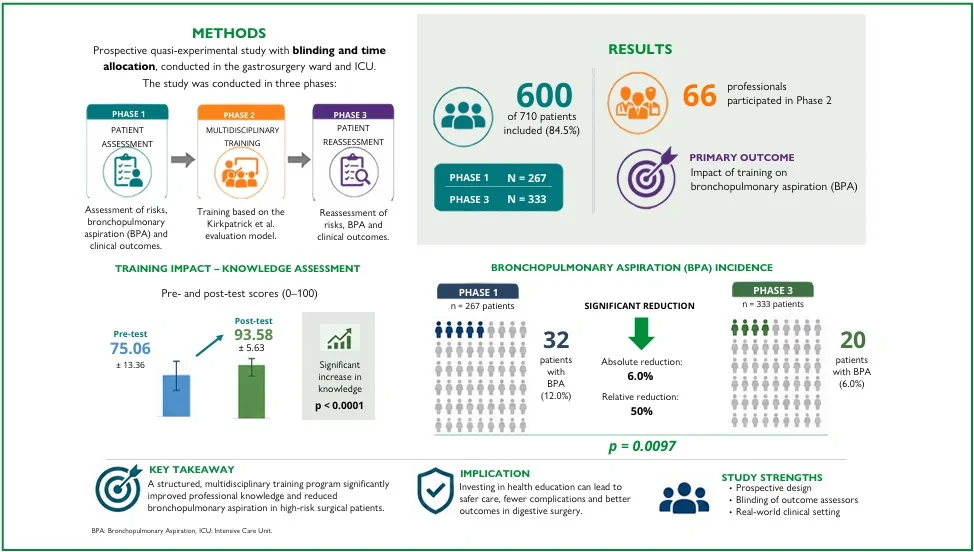

## RESUMO

**Racional:**

A aspiração broncopulmonar (ABP) ocorre quando alimentos ou fluidos atingem as vias aéreas inferiores, podendo causar hipóxia, pneumonia aspirativa e até morte. A prevenção exige identificação precoce dos riscos e atuação da equipe multiprofissional. A educação contínua da equipe, com protocolos, é essencial para reforçar medidas preventivas e melhorar os cuidados de saúde.

**Objetivos:**

Avaliar o impacto do treinamento, baseado em protocolo estabelecido, no aprendizado dos profissionais e na incidência de aspiração broncopulmonar.

**Métodos:**

Estudo prospectivo quasi-experimental, com cegamento e alocação temporal, realizado na enfermaria e UTI da Gastrocirurgia. Foi dividido em três fases: avaliação dos pacientes, capacitação multiprofissional baseado em protocolo bem estabelecido, e reavaliação dos pacientes quanto a riscos, aspiração broncopulmonar e desfechos.

**Resultados:**

O estudo incluiu 600 dos 710 pacientes avaliados, distribuídos em duas fases: Fase 1 (n=267) e Fase 3 (n=333). Participaram 66 profissionais na Fase 2. Avaliou-se o impacto de uma capacitação sobre aspiração broncopulmonar (ABP) por meio de avaliações pré e pós-treinamento. A pontuação aumentou significativamente (pré=75,06±13,36; pós=93,58±5,63; p<0,0001). Na Fase 1, 32 pacientes apresentaram ABP; na Fase 3, 20, diferença significativa (p=0,0097).

**Conclusões:**

A capacitação mostrou-se eficaz na prevenção da aspiração broncopulmonar e nos desfechos clínicos, com impacto positivo em todos os níveis do protocolo utilizado. Os resultados reforçam a educação continuada como estratégia para segurança do paciente e gestão de riscos, sugerindo sua aplicação em outros contextos assistenciais.

## INTRODUCTION

 The term “bronchoaspiration” or “bronchopulmonary aspiration” (BPA) refers to the entry of food particles, oral mucosal secretions, or gastric contents into the lower respiratory tract^4,9,10^. BPA affects three major demographic groups: children, older adults, and patients admitted to intensive care units (ICUs). Individuals with dysphagia, postoperative patients, and those with neurological impairment are also particularly vulnerable to this event^9,29^ . 

 BPA is considered the most significant cause of morbidity and mortality in these populations^9^. Approximately 50% of patients admitted to ICUs experience microaspiration^3^. BPA has been reported in 38% of patients with severe trauma^27^ and in 42 to 87% of patients undergoing prolonged orotracheal intubation^13^. Previous studies have reported that, among patients who experience cardiac arrest, 65% develop aspiration pneumonia, likely due to BPA occurring during cardiopulmonary resuscitation^20^. 

 The main risk factors associated with this adverse event include altered level of consciousness, reduced gastrointestinal motility, poor oral hygiene, invasive mechanical ventilation (IMV), enteral feeding tubes, dysphagia secondary to neurological disorders, prolonged vomiting, gastric obstruction, and aging^21,27^. In addition, other conditions may increase the risk of BPA, including seizures, stroke, intoxication, pharyngeal muscle injury, ineffective cough, anatomical abnormalities, use of sedative agents, head and neck surgery, and changes in body position^10^. 

 The risks associated with BPA are substantial, as aspirated material may partially or completely obstruct the airways, resulting in hypoxemia, pulmonary infections such as aspiration pneumonia, and even death in severe cases. Clinical signs of BPA may include sudden coughing during or after feeding, respiratory distress, expiratory wheezing, fever in the presence of secondary infection, and, in extreme cases, respiratory arrest^33^. 

 Complications associated with BPA are diverse and include pulmonary atelectasis, lung abscess, chemical pneumonitis, and acute respiratory distress syndrome, a severe inflammatory response of the lungs. The most common complication is aspiration pneumonia, which increases the risk of mortality and prolongs hospital length of stay, consequently resulting in higher healthcare costs^27^. 

 Therefore, preventing BPA and its associated complications requires the early identification of patients at risk for this adverse event, as well as the consistent implementation of preventive measures by the entire multidisciplinary healthcare team^27^. 

 BPA risk management involves the adoption of preventive and interventional measures, including assessment of the risk of this adverse event^3,22^. To prevent BPA, measures such as appropriate swallowing assessment, proper positioning during feeding, administration of food with a texture appropriate to the patient’s needs, and education of caregivers and healthcare professionals are essential. The implementation of rigorous protocols and continuous training is fundamental to minimizing the risk of aspiration^25^. 

 Education for multidisciplinary healthcare teams has a substantial impact and can be enhanced through continuous training, using models such as Kirkpatrick et al., which can help evaluate and improve the implementation of preventive measures in healthcare^5,11^. 

 The Kirkpatrick et al. training evaluation model is widely used to measure the effectiveness of educational programs and evaluate training initiatives. It comprises four levels of evaluation: Reaction, Learning, Behavior, and Results. In the context of BPA prevention, this model can be applied in several ways, as below^5,11^ :

- Level 1 – Reaction: Assesses healthcare professionals’ reactions to training on BPA preventive measures through immediate feedback following the training;
- Level 2 – Learning: Measures improvements in professionals’ knowledge of BPA signs, risk factors, and preventive strategies through pre- and post-training knowledge assessments;
- Level 3 – Behavior: Assesses changes in professionals’ behavior in clinical practice, such as the adoption of new feeding techniques and patient management strategies to reduce the risk of aspiration;
- Level 4 – Results: Analyzes reductions in the incidence of BPA and its complications following the implementation of preventive measures, directly linking training effectiveness to clinical outcomes.

Therefore, the objective of this study was to evaluate healthcare professionals’ learning outcomes following the proposed educational methodology, using the Kirkpatrick et al. protocol^5,11^, and to assess the impact of the training program on the incidence of BPA events among gastrointestinal surgery service patients. 

## METHODS

 This was a prospective, blinded, time-allocated, quasi-experimental study conducted with healthcare professionals and hospitalized patients in the ward and ICU of the University Hospital Digestive Surgery Unit, Universidade Estadual de Campinas, Campinas (SP).

- Inclusion criteria: hospitalized patients of both sexes and healthcare professionals affiliated with the hospital and members of the multidisciplinary team of the Digestive System Surgery Unit;
- Exclusion criteria: patients and healthcare professionals who declined to participate and/or did not sign the informed consent form, as well as those who were not affiliated with the Digestive Surgery Unit.

The study consisted of three phases:

- Phase 1: hospitalized patients were assessed for risk factors, BPA events, and clinical outcomes;
- Phase 2: the multidisciplinary healthcare team underwent training, with knowledge assessed before and after the training according to the Kirkpatrick et al. protocol^5,11^;
- Phase 3: hospitalized patients were assessed for risk factors, BPA events, and clinical outcomes.

### Data acquisition

 During Phase 1, patients were enrolled without being informed that they would participate in the study and before the healthcare team received training. Phase 2 consisted of training the healthcare professionals, who were unaware of the data collected from the patients included in Phases 1 and 3 of the study. In Phase 3, patients were enrolled after the healthcare team had completed training. Thus, blinding of both patients and healthcare professionals was ensured to preserve the integrity of the study and minimize bias. 

 In Phase 1, patients with and without risk factors for BPA were enrolled, and BPA events and clinical outcomes were monitored and assessed daily throughout hospitalization, until hospital discharge or death. Patients were assessed for the presence or absence of BPA risk using a predefined risk assessment table based on the literature, as well as for the occurrence of any BPA episode. 

 During this observation period, patient outcomes were assessed with regard to worsening respiratory status requiring supplemental oxygen therapy, orotracheal intubation, new infection diagnosed by the medical team, initiation of a new course of antibiotics prescribed by the medical team, length of hospital stay, hospital discharge, or death. 

 Phase 2 consisted of training the multidisciplinary healthcare team using specific tools to promote adherence to the educational methodology, as well as to assess learning and adherence according to the Kirkpatrick et al. protocol^5,11^. The multidisciplinary training consisted of didactic theoretical lectures, on-site availability for questions and clarification, and discussion of specific clinical cases. The participating professionals completed a questionnaire before training and two months after training to assess the teaching methodology and learning, according to the Kirkpatrick et al. protocol^5,11^. 

 In Phase 3, the trained healthcare professionals were informed about the patients included in the study, including those with and without risk factors for BPA, about whether BPA events occurred, and the corresponding clinical outcomes. Patients were monitored and assessed daily until hospital discharge or death, according to the previously established protocol. 

 Patient outcomes were assessed with regard to worsening respiratory status requiring supplemental oxygen therapy, orotracheal intubation, new infection diagnosed by the medical team requiring a new course of antibiotics prescribed by the medical team, length of hospital stay, hospital discharge, or death. Data from Phase 1 were then compared and correlated with those from Phase 3. 

### Statistical analysis

 Descriptive analysis was performed using frequency tables for categorical variables and measures of central tendency and dispersion for continuous variables. 

 For comparisons between the two groups, the MannWhitney U test was used for continuous variables, while the χ^2^ test or Fisher’s exact test was used for categorical variables. 

 Pre- and post-training comparisons were performed using the Wilcoxon signed-rank test. A significance level of 5% was adopted for all statistical analyses. 

## RESULTS

 A total of 710 patients were assessed, of whom 110 were excluded because they declined to participate in the study, did not sign the informed consent form, or were lost to follow-up by the Digestive Surgery Unit. 

 Thus, a total of 600 patients were included and divided into two groups: Phase 1, comprising 267 patients, and Phase 3, comprising 333 patients (Table 1). The mean age of the participants was 56.6 years (±14.6 years), with a median age of 60 years. The sex distribution was relatively balanced, with 46.8% (n=281) women and 53.2% (n=319) men. 

**Table 1:** Surgical procedures performed in Phases 1 and 3.

Surgical group	Phase 1 n (%)	Phase 3 n (%)
Hepatobiliary surgeries (hepatectomy, cholecystectomy, liver biopsy, biliodigestive bypass, liver transplantation)	125 (46.82)	203 (60.96)
Gastric and bariatric surgeries (gastrectomy, sleeve gastrectomy, fundoplication, gastric biopsy)	58 (21.72)	57 (17.12)
Pancreaticoduodenal surgeries (pancreatectomy, pancreaticoduodenectomy, pancreaticoduodenectomy with gastrectomy)	37 (13.86)	19 (5.71)
Esophageal surgeries (esophagectomy, esophagogastric fundoplication, esophagocoloplasty, esophageal dilation)	17 (6.37)	21 (6.31)
Colorectal and intestinal surgeries (rectosigmoidectomy, colectomy, intestinal transit reconstruction)	9 (3.37)	3 (0.90)
Other surgeries^*^	21 (7.87)	30 (9.01)
Total	267 (100)	333 (100)

* Other surgeries: herniorrhaphy, exploratory laparotomy, splenectomy, pharyngectomy, endoscopy, jejunostomy, gastrostomy, and other low-frequency procedures.

 A total of 80 healthcare professionals participated in the study, of whom 14 were excluded because they declined to participate, did not sign the informed consent form, or did not complete the pre- and/or post-training questionnaires. Thus, 66 professionals were included, of whom 56 (84.85%) were female, and 10 (15.15%) were male. The multidisciplinary team comprised 17 nurses (25.76%), 12 physiotherapists (18.18%), 2 physicians (3.03%), and 35 nursing technicians (53.03%). 

### Technical training of the multidisciplinary team

 The study evaluated the impact of professional training on BPA through a pre- and post-training assessment to measure the effectiveness of the training program: mean and standard deviation (SD). The results of the overall scores, individual questions, and their statistical significance are presented below:

- Pre-training score: Mean=75.06 (SD=13.36), Score range=35.90 to 88.90;
- Post-training score: Mean=93.58 (SD=5.63), Score range=65.20 to 100.00.

The difference between the pre- and post-training mean scores was statistically significant (p<0.0001), indicating a substantial improvement in the knowledge of the participating healthcare professionals following the training provided in Phase 2 of the study. 

 The results for the individual questionnaire items also demonstrated significant improvements: 

#### Question 1 (What is BPA?):

- Pre-training score: Mean=80.30 (SD=40.08), Score range=0.00 to 100.00;
- Post-training score: Mean=96.97 (SD=17.27), Score range=80.00 to 100.00.

*Significance: p=0.0010 (p<0.05), indicating a significant improvement in knowledge regarding the topic assessed. 

#### Question 2 (What are the signs of BPA?):

- Pre-training score: Mean=69.00 (SD=12.00);
- Post-training score: Mean=85.80 (SD=9.27).

*Significance: p<0.0001, demonstrating a significant improvement. 

#### Question 3 (What are the risks of BPA?):

- Pre-training score: Mean=75.08 (SD=15.01);
- Post-training score: Mean=98.22 (SD=4.85).

*Significance: p<0.0001, indicating a significant improvement. 

#### Question 4 (What are the preventive measures for BPA?):

- Pre-training score: Mean=75.83 (SD=11.82);
- Post-training score: Mean=93.26 (SD=7.15).

*Significance: p<0.0001, indicating a significant improvement. 

### Bronchopulmonary aspiration risk

 The analysis of BPA risk revealed that 521 of the patients studied (86.8%) were at risk of BPA, with no significant difference between the phases (p=0.9700, p>0.05). The most prevalent risk factors in this group were gastrointestinal risks, such as abdominal distension, nausea, and vomiting (358), followed by nutritional risk (186). Patients at risk of aspiration (86.8% of the total) were more likely to experience the event (p=0.0033, p<0.05 for the overall sample, and p=0.0116, p<0.05, in Phase 1). 

 The analysis of the association between BPA risk and the occurrence of aspiration revealed that none of the patients who were not considered at risk experienced BPA. 

 In Phase 1, 32 patients experienced BPA, compared with 20 patients in Phase 3 (p=0.0097, p<0.05). 

 The correlation between BPA data in Phases 1 and 3 can be analyzed based on the information presented in Table 2. 

**Table 2:** Bronchopulmonary Aspiration Data for Phases 1 and 3.

Phase	Bronchopulmonary Aspiration (%)	No Bronchopulmonary Aspiration (%)	Total	p-value
Phase 1	32 (12)	235 (88)	267	0.0097
Phase 3	20 (6)	313 (94)	333	
Total	52 (8.7)	548 (91.3)	600	-

 Effectiveness of the Intervention: The reduced incidence of BPA in Phase 3 (6 vs. 12% in Phase 1) suggests that multidisciplinary training can be life-saving, with a number needed to treat (NNT) of 17 to prevent one case of BPA (Table 3). 

**Table 3:** Analytical summary of intervention effectiveness.

Variable	Phase 1 (n=267) (%)	Phase 3 (n=333) (%)	Reduction (%)	p-value	NNT
BPA incidence	12	6	50	0.0097	17
GI risks	71.6	66.4	-	0.2107	-

NNT: number needed to treat=1/(0.12−0.06)=17 patients needed to be treated to prevent one case of BPA; GI: gastrointestinal; BPA: bronchopulmonary aspiration.

### Length of hospital stay

 Length of hospital stay was analyzed by comparing patients who experienced BPA with those who did not (Table 4). 

**Table 4:** Relationship between length of hospital stay and bronchopulmonary aspiration in phases 1 and 3.

Phase	Group	Total	Length of Hospital Stay (mean/days)	Standard Deviation (days)	Median (days)	Range (days)	P-value
Phase 1	No Bronchopulmonary Aspiration	235	8.6	7.7	6.0	1.0–52.0	<0.0001
	Bronchopulmonary Aspiration	32	24.9	28.3	13.0	3.0–129.0	
Phase 3	No Bronchopulmonary Aspiration	313	8.1	11.1	5.0	0.0–130.0	<0.0001
	Bronchopulmonary Aspiration	20	40.1	43.2	23.5	5.0–162.0	

### Clinical outcomes

 Among the 600 patients analyzed, 553 were discharged from the hospital (92.2%), while 47 died (7.8%). Among patients who experienced BPA, the mortality rate was significantly higher: 12 of the 52 patients (23.1%) died, as shown in Table 5. In contrast, among patients who did not experience BPA, the mortality rate was only 6.4% (35 of 548 patients). 

**Table 5:** Comparison of clinical outcomes according to the presence of bronchopulmonary aspiration.

Outcome	Bronchopulmonary Aspiration (%)	No Bronchopulmonary Aspiration (%)	Total
Hospital discharge	40 (76.9)	513 (93.6)	553
Death	12 (23.1)	35 (6.4)	47
Total	52	548	600

 The presence of BPA was associated with a significantly higher risk of death, both in Phase 1 (54.5% of patients who died vs. 10.2% of those who were discharged) and Phase 3 (16.7 vs. 4.7%), with p=0.0132 (p<0.05). 

 The most common complications among these patients were the need for a new course of antibiotics (68.75% in Phase 1; 42.86% in Phase 3) and orotracheal intubation (18.75% in Phase 1; 28.57% in Phase 3). Importantly, all deaths occurred among patients who were at risk of aspiration (p=0.0014, p<0.05), highlighting the severity of this complication. 

### Kirkpatrick et al. learning evaluation model

 The analysis of the association between the four evaluation levels—reaction, learning, behavior, and results—and the findings of this study yielded the following results. 

### Level 1 – reaction (participant satisfaction)

 The results for Level 1 demonstrated high acceptance of the training program among the multidisciplinary team. Participants evaluated the training using a 5-point Likert scale^14^ across two main domains:

- Satisfaction with the training and perceived importance of the content: 85% of the 66 participants assigned a score of 5 (“Very important”), while 5% assigned a score of 4 (“Important”). Only a minority (7%) provided scores ≤3, indicating a strong overall consensus regarding the relevance of the training content;
- Perceived benefits for patients and the institution: Similarly, 85% of participants rated the impact as 5 (“Very important”), while only 5% assigned scores ≤2, reinforcing that most participants recognized the practical value of the professional training.

### Level 2: learning (knowledge acquisition)

 A significant increase was observed in post-training assessment scores compared with pre-training scores (75.06 vs. 93.58; p<0.0001). Improvement was observed across all specific questions (e.g., Question 3: 75.08 vs. 98.22; p<0.0001). 

 Conclusion: The training program was effective in promoting the acquisition of theoretical knowledge. 

### Level 3: behavior (application in clinical practice)

 A reduction in the incidence of BPA was observed (Phase 1: 12.0% vs. Phase 3: 6.0%; p=0.0097, p<0.05), along with changes in risk assessment practices among healthcare professionals. 

 Conclusion: The findings indicate a behavioral change, characterized by the adoption of safer clinical practices. 

### Level 4: results (organizational and clinical impact)

 A reduction in complications was observed, with fewer cases of BPA associated with orotracheal intubation and antibiotic therapy in Phase 3. Patients who experienced BPA had longer hospital stays (p<0.0001) and higher mortality rates (p=0.0003, p<0.05). The reduction in BPA observed in Phase 3 may have contributed to improved clinical outcomes. 

## DISCUSSION

 The present study evaluated 600 patients undergoing gastrointestinal surgery, divided into two phases (Phase 1: n=267; Phase 3: n=333), with a mean age of 56.6 years (±14.6 years) and a balanced sex distribution (46.8% women and 53.2% men). These demographic characteristics are consistent with those reported in the literature, particularly the findings of Machlowska et al.^15^, who reviewed the epidemiology, risk factors, classification, genomic characteristics, and treatment strategies for gastric cancer, as well as those of the multicenter study by Szakmany et al.^26^, which evaluated in-hospital clinical outcomes in patients undergoing upper gastrointestinal tract surgery. Overall, our findings are consistent with the existing literature with regard to the demographic characteristics of the study population. 

 Among the 66 healthcare professionals who participated in the training program, there was a predominance of women, with 56 participants (84.85%). These findings reflect the typical profile of healthcare teams in high-complexity hospital settings in Brazil, where female nursing professionals constitute the largest occupational group and men remain underrepresented. According to the World Health Organization, women account for approximately 90% of the global nursing workforce^8^. Therefore, the composition of the team in our study is representative of a high-complexity university hospital, with a gender and professional distribution broadly consistent with the global healthcare workforce. This supports the external validity and potential generalizability of our findings regarding training uptake and effectiveness, particularly in the context of BPA prevention. 

 Furthermore, the multidisciplinary training resulted in a substantial improvement in knowledge regarding BPA, with the overall score increasing from 75.1±13.4 to 93.6±5.6 points (p<0.0001). These gains are consistent with evidence from the literature using pre- and post-training assessments to evaluate the effectiveness of healthcare training programs. Kunzler et al.^12^ demonstrated that an educational program on ventilator-associated pneumonia increased the proportion of correct responses from 62% in the pre-training assessment to 84% in the post-training assessment in an ICU team. 

 Recent studies by Yang et al.^32^ evaluating nursing training programs also reported significant improvements in theoretical knowledge and practical skills (p<0.05), likewise using the Kirkpatrick et al. protocol^5, 11^. 

 The increase of approximately 18–22 percentage points following the intervention, consistent with the findings of the present study, further supports the effectiveness of the educational strategies employed. The use of specific assessments addressing the definition, signs, risk factors, and preventive measures of BPA, with p=0.001 (p<0.05), indicates substantial learning of key concepts. These findings are consistent with reviews supporting the use of pre- and post-training assessments in health education (Kirkpatrick et al.^5,11^), as they provide objective measures of cognitive gain and educational effectiveness. This strengthens the internal validity of the study and suggests that well-structured training programs can be effective across different clinical contexts, including BPA prevention^1,14^. 

 The results also corroborate current guidelines and recommendations (WHO^31^; ANVISA^3^), which emphasize the importance of continuing education in patient safety, particularly concerning preventable adverse events such as BPA. The consistency of these findings suggests that the training model was effective in expanding the theoretical knowledge of the healthcare team, which is essential for improving clinical practice^14^. 

 According to Zhao et al.^34^, a study evaluating the effectiveness of a training program for healthcare professionals to identify aspiration pneumonia risk factors demonstrated a significant improvement in professionals’ knowledge following the intervention, strengthening their ability to recognize relevant clinical conditions. These findings are consistent with those of the present study. 

 In the present study, risk factors for BPA were identified in 86.8% of patients, with a strong association between the presence of risk factors and the occurrence of the event (p=0.0033, p<0.05). These findings are supported and further contextualized by two relevant systematic reviews. Tang et al.^28^ analyzed the incidence and risk factors for aspiration pneumonia following endoscopic submucosal dissection for gastric lesions. Their findings highlighted gastrointestinal functional disturbances, including gastroesophageal reflux, nausea, and vomiting, as factors significantly associated with aspiration. This is consistent with the present study, in which gastrointestinal symptoms represented the most prevalent risk category. 

 Furthermore, the finding that none of the participants without identified risk factors developed BPA reinforces the potential value of clinical risk assessment and suggests good predictive performance of the criteria used in this study. 

 STARSurg Collaborative and TASMAN Collaborative^24^ evaluated prognostic models for postoperative pulmonary complications following major abdominal surgery, including aspiration among the clinical outcomes assessed. Their analysis showed that many existing models lack accuracy in predicting events such as BPA, particularly when gastrointestinal and nutritional factors are not systematically incorporated. In this context, the findings of the present study provide evidence that clinical variables, such as abdominal distension and nutritional risk, may be sensitive predictors of BPA. 

 When compared with the existing literature, the findings of this study demonstrate a consistent recognition of the importance of gastrointestinal factors as critical BPA prevention determinants. Furthermore, the high prevalence of identified risk factors highlights the need for systematic screening and early intervention strategies, which are not yet fully incorporated into commonly used prognostic models, as indicated by the literature reviewed. 

 Based on the findings of the present study, a statistically significant reduction in the incidence of BPA was observed between Phases 1 and 3, decreasing from 12 to 6% (p=0.0097, p<0.05). This downward trend is consistent with the recent literature emphasizing the importance of identifying risk factors and implementing preventive measures early. 

 Mandell and Niederman^16^ provided a comprehensive overview of aspiration pneumonia, emphasizing that aspiration of gastric contents is frequently underdiagnosed and undertreated despite its clinical relevance. The authors highlighted prophylactic interventions and monitoring of risk factors as key strategies for reducing the occurrence of aspiration-related complications. These findings support the effectiveness of the measures implemented between the study phases, which may have contributed to the significant reduction in BPA cases observed in Phase 3. 

 Perlas and Arzola^18^ reviewed strategies for mitigating aspiration risk, particularly in perioperative settings and among critically ill patients. The authors indicated that interventions such as appropriate fasting, the use of acid-suppressive medications, proper patient positioning, and preoperative risk assessment may significantly reduce aspiration events. This approach is consistent with the findings of the present study, suggesting that more rigorous preventive practices or changes in the protocol between study phases may have influenced the observed outcome. 

 Warner et al.^30^ analyzed closed-claim aspiration cases from medical liability databases and identified recurrent failures in screening and prevention protocols and inadequate documentation as organizational risk factors. In comparison with the findings of the present study, recognizing BPA as a potentially preventable outcome that is sensitive to the quality of care may have contributed to institutional improvements in clinical practices between the study phases, potentially explaining the significant incidence reduction observed between Phase 1 and Phase 3. 

 Therefore, the comparison between the findings of the present study and the existing literature demonstrates clear convergence: the risk of BPA is high but preventable through appropriate clinical and organizational interventions. The reduction in cases observed between the study phases is consistent with evidence-based recommended strategies, indicating that the measures implemented between the phases were effective and should be maintained or further expanded. 

 The results of this study demonstrated that patients who experienced BPA had a significantly longer hospital stay than those who did not, both in Phase 1 (24.9 vs. 8.6 days; p<0.0001) and Phase 3 (40.1 vs. 8.1 days; p<0.0001). These findings reinforce the existing literature, which identifies BPA as an important factor associated with increased hospital morbidity and prolonged hospitalization. Mandell and Niederman^16^ highlighted that BPA is strongly associated with longer hospital stays, the need for prolonged ventilatory support, and increased rates of respiratory infectious complications. 

 Furthermore, Perlas and Arzola^18^, in their discussion of BPA in the anesthetic setting, emphasized that BPA, even when recognized early, can increase hospital length of stay and intensive care costs. Similarly, the analysis conducted by Warner et al.^30^ demonstrated that, among documented BPA cases in North American centers, the complication was associated with more severe outcomes and longer hospital stays, and was considered one of the perioperative events with the greatest clinical and financial impact. 

 The findings of the present study corroborate the existing literature by demonstrating that BPA is associated with a significant increase in hospital length of stay. The longer hospital stay observed in Phase 3 (40.1 vs. 24.9 days in Phase 1) may reflect differences in disease severity or the presence of additional comorbidities. 

 In the present study, overall mortality was 7.8% (47/600), while among patients who experienced BPA, the mortality rate reached 23.1% (12/52), compared with only 6.4% among patients without BPA (35/548; p=0.013, p<0.05). These findings underscore the clinical severity of BPA and are consistent with several international studies reporting relevant mortality rates. A recent Canadian study by Shin et al.^23^ reported an in-hospital mortality rate of 27.8% among patients aged ≤65 years with aspiration pneumonia, a rate comparable to that observed in older adults with aspiration pneumonia. 

 In a larger cohort encompassing more than 4,200 hospitals, Bai et al.^2^ reported a 30-day mortality rate of 29.4% among patients with aspiration pneumonia, compared with 11.6% among patients without aspiration pneumonia. 

 Additionally, Putot et al.^19^ reported a 30-day mortality rate of up to 31% following aspiration pneumonia, compared with 15% among patients with non-aspiration pneumonia. 

 These studies reinforce that the fatal outcome observed in the present study — 23.1% among patients with BPA — is lower but remains consistent with estimates reported in large international cohorts (27–31%). It is noteworthy that the significantly higher mortality among patients with BPA compared with those without BPA further supports the association between the absence of this event and more favorable clinical outcomes, as demonstrated by hospital discharge rates of 93.6% among patients without BPA versus 76.9% among those who experienced BPA. 

 Level 1 evaluation (Reaction/Satisfaction) demonstrated high participant satisfaction with the training program, consistent with the Kirkpatrick et al. protocol^5, 11^, in which satisfaction in Level 1 may facilitate the transfer of learning and contribute to subsequent outcomes. According to Eid and Quinn^6^, participant satisfaction facilitates the practical application of acquired knowledge. Similarly, Muñoz-Castro et al.^17^ reported that factors such as content relevance, training format, and duration positively influenced healthcare professionals’ perceptions. In the present study, most participants assigned the highest ratings to training content importance and its impact on clinical practice. In-person, brief training activities with certification were associated with higher satisfaction, reinforcing the preference for formats that emphasize practical applicability and interaction^7,17^. 

 Regarding Level 2 (Learning), this study demonstrated a statistically significant increase in knowledge assessment scores from pre- to post-training, supporting the effectiveness of the intervention in promoting the acquisition of theoretical knowledge. Similarly, in the review by El Nsouli et al.^7^, most of the studies assessed achieved this level, indicating that educational interventions contributed to improving participants’ knowledge and attitudes. 

 At Level 3 (Behavior), the present study provides concrete evidence of changes in clinical practice, including a reduction in the incidence of BPA and changes in risk assessment following the training intervention, supporting the effectiveness of the program in promoting behavioral change in clinical practice. In contrast, the systematic review found that few studies reached or evaluated this level, highlighting the need for longitudinal studies with greater methodological rigor to establish sustained changes in professional behavior and their impact on actual clinical practice^7^. 

 At Level 4 (Results), the present study was able to associate the training intervention with relevant clinical outcomes, including a reduction in complications, length of hospital stay, and mortality rates, demonstrating an effect that extends beyond individual knowledge improvement to broader organizational and patient-safety outcomes. The systematic review by El Nsouli et al.^7^ identified an important gap at this level, with few studies demonstrating long-term effects and tangible clinical impact, largely because of the scarcity of longitudinal studies and the methodological challenges involved in measuring organizational and broader health outcomes. 

 Evidence of changes in professional behavior and clinical outcomes reinforces the importance of comprehensive evaluation models, such as those proposed by Kirkpatrick et al.^5, 11^, with an emphasis on longitudinal studies exploring the relationship between training, changes in professional practice, and patient health outcomes. The present study contributes to the literature by demonstrating not only participant satisfaction and immediate learning gains, but also evidence of behavioral change and clinical impact. These findings highlight the need to invest in continuing professional education that extends beyond immediate reactions and evaluates tangible outcomes in clinical practice. 

## CONCLUSIONS

 This study clearly demonstrated, with statistically significant findings, that structured multidisciplinary training is effective in preventing BPA and improving clinical outcomes in a university hospital setting. Using the Kirkpatrick et al. protocol^5, 11^, the results demonstrated positive effects across all evaluated levels, supporting the premise that investment in continuing education can strengthen patient safety, reduce complications, and optimize hospital resources. 

 The findings reinforce the importance of well-designed educational interventions as a strategic tool for improving healthcare quality. Furthermore, they provide a solid basis for implementing institutional policies focused on risk management and the prevention of adverse events. Future research should evaluate the application of this training model in different clinical settings, thereby expanding its impact on clinical practice. 

## Data Availability

The datasets generated and/or analyzed during the current study are available from the corresponding author upon reasonable request.
